# Life-course fertility and multimorbidity among middle-aged and elderly women in China: Evidence from China health and retirement longitudinal study

**DOI:** 10.3389/fpubh.2023.1090549

**Published:** 2023-02-20

**Authors:** Mingjun Chen, Jianhui Guo, Yawen Lin, Jialiang Xu, Yuduan Hu, Le Yang, Xingyan Xu, Li Zhu, Jungu Zhou, Zhiyu Zhang, Huangyuan Li, Shaowei Lin, Siying Wu

**Affiliations:** ^1^Department of Epidemiology and Health Statistics, School of Public Health, Fujian Medical University, Fuzhou, China; ^2^School of Public Health, Fujian Medical University, Fuzhou, China; ^3^Department of Preventive Medicine, School of Public Health, Fujian Medical University, Fuzhou, China

**Keywords:** multimorbidity, life course, women health, aging, fertility history

## Abstract

**Background:**

Multimorbidity has become an important public health problem in China, especially among middle-aged and elderly women. Few studies have been reported on the association between multimorbidity and female fertility, which is an important stage in the life course. This study aimed to explore the association between multimorbidity and fertility history among middle-aged and elderly women in China.

**Methods:**

Data from 10,182 middle-aged and elderly female participants in the China Health and Retirement Longitudinal Study (CHARLS) in 2018 were used in this study. Multimorbidity was defined as the presence of at least two or more chronic conditions. Logistic regression analysis, negative binomial regression analysis, and restrictive cubic splines (RCSs) were used to analyze the relationship between female fertility history and multimorbidity or the number of chronic conditions. Multivariable linear regression was used to analyze the relationship between female fertility history and multimorbidity pattern factor scores.

**Results:**

The results of this study showed that high parity and early childbearing were significantly associated with an increased risk of multimorbidity and an increased number of chronic conditions among middle-aged and elderly women in China. Late childbearing was significantly associated with reduced risk of multimorbidity and lessened diseases. Parity and age of first childbirth were significantly correlated with the odds of multimorbidity. The association between fertility history and multimorbidity was found to be influenced by age and urban–rural dual structure. Women with high parity tend to have higher factor scores of cardiac-metabolic, visceral-arthritic, and respiratory-psychiatric patterns. Women with early childbearing tended to have higher factor scores of the visceral-arthritic pattern and those with late childbearing tended to have lower factor scores of the cardiac-metabolic pattern.

**Conclusion:**

Fertility history has a significant effect on multimorbidity in the middle and later lives of Chinese women. This study is of great importance for reducing the prevalence of multimorbidity among Chinese women through their life course and promoting health during their middle and later lives.

## 1. Introduction

Multimorbidity is defined as a simultaneous occurrence of two or more chronic diseases in one person at a certain time, including physical and mental health complications (1). In general, multimorbidity is one of the inevitable outcomes driven by the accumulation of critical events in a longitudinal life course according to life-course theories (2). Studies have consistently linked multimorbidity to lower quality of life (3), higher financial burdens (4, 5), greater psychological stress (6, 7), and higher mortality (8) compared to single diseases. Given the unprecedented aging of the population in China, multimorbidity is regarded as a prominent health problem among Chinese middle-aged and elderly adults, with a prevalence rate of nearly 50% (9, 10). How to effectively and accurately predict the rising prevalence of multimorbidity caused by the rapid aging process has become an important public health challenge in China.

Previous studies have shown sex inequalities in multimorbidity. Women tend to face a higher risk of multiple chronic diseases than men (11). In China, middle-aged and elderly women are more vulnerable to multimorbidity, and they suffer from an increased mental health risk due to their biological characteristics and socioeconomically disadvantageous situations (10, 12). However, the current focus on women's health is still limited to sexual and reproductive health, with no importance attached to their dilemma of facing additional sex-specific risk factors beyond the traditional risk factors of multimorbidity (13).

Female fertility is an important stage in women's life course, including indices such as parity and childbearing age. Some evidence from Western countries has suggested that women's childbearing history has a significant effect on mid-late life health. For instance, women with high parity and those with premature childbearing history are more likely to experience poorer health outcomes and higher mortality in their later life (14–16). On the contrary, late childbearing has been reported to have a significant association with better cognitive function (17). Due to the profound tradition of “family culture” in China, fertility history is particularly important to Chinese women's life course (18). The results of existing reports on the female fertility and health of Chinese women in their middle and later lives are similar to those in Western countries. Elderly women with high parity tend to suffer from activities of daily-living impairment and poorer self-rated health (19). Early age of first childbirth is associated with a higher risk of cardiovascular diseases (20). Women who had been able to have children after the age of 35 years are more likely to have a longer life (21).

The focus must be shifted from single diseases and childbearing events in specific periods to multimorbidity and complete fertility history in the life course to improve the health of women in their middle and later lives. At present, no related literature could be found on the association between fertility history and multimorbidity, not to mention when the subject is middle-aged and elderly women in China.

By using the data from China Health and Retirement Longitudinal Study (CHARLS) conducted in 2018, the study aimed to explore the relationship between life-course fertility and multimorbidity among middle-aged and elderly women in China. Subgroup analyses and tests for interaction were conducted to further evaluate whether the associations were modified by age and region. In addition, the effect of fertility on the multimorbidity pattern factor scores was investigated.

## 2. Materials and methods

### 2.1. Data

The data used in this study was from China Health and Retirement Longitudinal Study (CHARLS) conducted in 2018. CHARLS is a nationally representative longitudinal study of the older population of China, covering 28 provinces, 150 counties (districts), and 10,624 families. CHARLS employed stratified probability proportional to size random sampling principles to make sure the representativeness of samples. The baseline survey was conducted in 2011, followed up in 2013 (wave 2), 2015 (wave 3), and 2018 (wave 4).

CHARLS 2018 survey was conducted from February to September 2018 and released in 2020. Of the total 19,816 individuals who participated in the CHARLS 2018 survey, 255 were younger than 45 years, 11,880 were between 45 years and 64 years, and 7,681 were 65 years or older. After excluding male participants, 10,475 female participants remained. After further excluding the participants younger than 45 years of age, and those with missing chronic diseases and fertility history data, 10,228 participants remained. Subsequently, 46 participants without biological children were excluded. Therefore, 10,182 female respondents were identified for analysis. The selection process of participants in this study is shown in Supplementary Figure 1.

### 2.2. Identification of chronic conditions and multimorbidity

Each participant was asked if they had been diagnosed with any of the following 14 chronic conditions including hypertension, diabetes, cancer, chronic lung disease, heart disease, stroke, psychiatric disease, arthritis, dyslipidemia, liver disease, kidney disease, digestive disease, asthma, and memory-related disease. When participants have ever been diagnosed with two or more of the 14 aforementioned conditions, they were regarded as having multimorbidity.

### 2.3. Multimorbidity pattern factor score

Exploratory factor analysis was used to determine how chronic conditions tend to gather together to exhibit multimorbidity patterns among middle-aged and elderly women in China (22). Kaiser–Meyer–Olkin method and Bartlett test of sphericity were used to estimate the adequacy of the sample in factor analysis. Factors were extracted using the principal factor method based on tetrachoric correlation matrices. The number of factors was identified based on their interpretability, eigenvalue, and scree plot shape. Factor interpretation was facilitated with an oblique rotation (Oblimin) of factor-loading matrices. For determining the most appropriate multimorbidity patterns, chronic conditions with a factor loading above 0.20 were selected. Multimorbidity patterns were named according to common features of diseases in the patterns. Factor scores of each multimorbidity pattern were used to assess each participant's multimorbidity status more specifically. To obtain factor scores, the factor loading of each chronic disease was multiplied by 1 (presence of chronic diseases) or 0 (absence of chronic diseases), then each item was summed to calculate each participant's total score, and normalized to the mean value of 0 and standard deviation of 1 (23, 24).

### 2.4. Female fertility history

Several important variables representing fertility history were collected by interviewing participants in the CHARLS 2018 survey: parity, age of first childbirth, and age of last childbirth. Parity refers to the number of biological children of the respondents, including deceased children. It was divided into three groups: having one child, having two children, and having three or more children. The age of early childbearing was set at 21 years after considering the current fertility status of Chinese women (19). The age of first childbirth was categorized as 21 years or above and younger than 21 years. If a woman gave her first childbirth before the age of 21 years, she was considered as having a history of early childbearing. Similarly, taking into account the fact that the age of 35 years and older were recognized as expectant mothers of older ages in China, the age of last childbirth was categorized as less than 35 years and 35 years or above (21). Women who gave their last birth at the age of 35 years or older were defined as late childbearing.

### 2.5. Covariates

General demographic characteristics, lifestyle and health behaviors, early physical conditions, and childhood socioeconomic conditions were controlled in this study. General demographic included age, marital status, educational level, socioeconomic status, and place of residence. Taking 65 years old as the standard for the division of middle-aged and elderly people in China, the age groups were divided into middle-aged women aged 45–64 years and elderly women aged 65 years or above. Marital status was categorized as married and other marital statuses, such as single, widowed, divorced, and separated. Educational level was based on the highest educational level achieved and categorized as elementary education or below and secondary education or above. Socioeconomic status was reflected by annual per-capita household expenditure, and defined as four levels based on quartiles of annual per-capita household expenditure. Place of residence, instead of hukou, was used to judge whether participants were from an urban community or rural village because residence could better reflect the socioeconomic environment around the participants. Lifestyle and health behaviors included smoking, drinking, and physical activities. Participants were asked if they had ever smoked cigarettes or drank alcoholic beverages to determine whether they smoked or drank. Physical activities were collected by asking participants whether they regularly engaged in physical activities. Early physical conditions included the age of menarche and childhood health status, collected by asking female participants their age of first menstruated period and health status in childhood compared with peers. The age of menarche was categorized as aged 15 years or younger and aged over 15 years. Childhood health status was categorized as about average, healthier, and less healthy. Childhood socioeconomic conditions were judged by asking participants about their families' financial situation compared to average families in their community or village at that time.

### 2.6. Statistical analysis

The chi-square test was used to assess differences in sample characteristics between groups. A variance inflation factor (VIF) was used to assess multicollinearity among the covariates adjusted for in our analysis. VIF of covariates in our analysis were all < 2, indicating that the assumption of reasonable independence among variables was met.

Multivariable logistic regression models and multivariable negative binomial regression models were used to analyze the relationship between female fertility history with multimorbidity and the number of chronic conditions, respectively. Multivariable negative binomial regression models were chosen instead of multivariable Poisson regression models because the number of chronic conditions was overdispersed (the variance was greater than the mean in the outcome variable). Models were adjusted for age, marital status, educational level, socioeconomic status, place of residence, smoking, drinking, physical activities, age of menarche, childhood health status, and childhood socioeconomic conditions. The results of the logistic regression analyses were presented as odds ratio (OR) and 95% confidence interval (95% CI), and the results of the negative binomial regression analyses were presented as an incidence rate ratio (IRR) and 95% CI. Due to China's obvious age stratification and special urban–rural dual structure, subgroup analyses were performed and stratified by age and residence to determine whether the association between female fertility history and multimorbidity differed among subgroups. A likelihood ratio test (LRT) was conducted to analyze the interactions by comparing models with interaction terms to those without interaction terms. A cutoff of *P* < 0.05 was used for significance.

Multivariable linear regression models were used to analyze the relationship between female fertility history and multimorbidity pattern factor scores. Models were adjusted for age, marital status, educational level, socioeconomic status, place of residence, smoking, drinking, physical activities, age of menarche, childhood health status, and childhood socioeconomic conditions. The results of the linear regression analyses were presented as β coefficients and a 95% confidence interval (95% CI).

Restrictive cubic splines (RCSs) were used to assess linear or nonlinear associations among female fertility (parity and age of first childbirth), multimorbidity, and factor scores. To better fit models, the node number of RCS is set to four. In the RCS model assessing the association between parity and multimorbidity, having one child was used as a reference. In the RCS model assessing the association between age of first childbirth and multimorbidity, 21 years was used as a reference. RSC models were adjusted for age, marital status, educational level, socioeconomic status, place of residence, smoking, drinking, physical activities, age of menarche, childhood health status, and childhood socioeconomic conditions.

Two sensitivity analyses were performed. First, to identify the effect of missing data on the robustness of the results, a random forest-based multiple imputation method by chained equations (MICE) was performed to fill in missing data and then re-analyze. On the assumption of missing data at random (MAR), the missing data were imputed for covariates with 20 iterations, and five datasets were generated (25). In addition, to evaluate the effect of rural and urban migration, hukou was used as a variable instead of residence to conduct a re-analysis.

Analyses in the study were conducted using SPSS version 26.0 and R version 4.2.1. LRT was performed using the “epicalc” package and RCS was performed using the “rms” package.

## 3. Results

### 3.1. Descriptive characteristics of the study participants

The descriptive characteristics of the study participants are shown in Table 1. Of the 10,182 women who participated included in the study, 5,965 (58.6%) had multimorbidity and 4217 (41.4%) were not. Statistical differences were observed in parity, age of first childbirth, age of last childbirth, age, marital status, educational level, hukou, smoking, and age of menarche between women with multimorbidity and those with no multimorbidity (*P* < 0.05).

**Table 1:** Descriptive characteristics of participants in this study.

**Variables**	**Total (*N =* 10182)**	**Multimorbidity**		***P***
		**No (*****N** =* **4217)**	**Yes (*****N** =* **5965)**	
**Parity**, ***n*** **(%)**				< 0.001
1	1688 (16.6)	870 (20.6)	818 (13.7)	
2	3779 (37.1)	1720 (40.8)	2059 (34.5)	
≥3	4715 (46.3)	1627 (38.6)	3088 (51.8)	
**Age of first childbirth**, ***n*** **(%)**				< 0.001
≥21 years old	7892 (77.5)	3399 (80.6)	4493 (75.3)	
< 21 years old	2290 (22.5)	818 (19.4)	1472 (24.7)	
**Age of last childbirth**, ***n*** **(%)**				< 0.001
< 35 years old	8726 (85.7)	3675 (87.1)	5051 (84.7)	
≥35 years old	1456 (14.3)	542 (12.9)	914 (15.3)	
**Age**, ***n*** **(%)**				< 0.001
45-64 years old	6275 (61.6)	3101 (73.5)	3174 (53.2)	
≥65 years old	3907 (38.4)	1116 (26.5)	2791 (46.8)	
**Marital status**, ***n*** **(%)**				< 0.001
Others	2615 (25.7)	936 (22.2)	1679 (28.1)	
Married	7567 (74.3)	3281 (77.8)	4286 (71.9)	
**Educational level**, ***n*** **(%)**				0.040
Elementary education or below	7890 (77.5)	3225 (76.5)	4665 (78.2)	
Secondary education or above	2292 (22.5)	992 (23.5)	1300 (21.8)	
**Socioeconomic status**, ***n*** **(%)**				0.119
Quartile 1	2105 (25.0)	889 (25.6)	1216 (24.6)	
Quartile 2	2102 (25.0)	900 (25.9)	1202 (24.3)	
Quartile 3	2099 (25.0)	831 (23.9)	1268 (25.7)	
Quartile 4	2104 (25.0)	852 (24.5)	1252 (25.4)	
**Place of residence**, ***n*** **(%)**				0.282
Urban community	4121 (40.5)	1733 (42.1)	2388 (40.0)	
Rural village	6061 (59.5)	2194 (58.9)	3577 (60.0)	
**Hukou**, ***n*** **(%)**				< 0.001
Agricultural hukou	7409 (72.8)	2977 (70.6)	4432 (74.3)	
Non-agricultural hukou	2773 (27.2)	1240 (29.4)	1533 (25.7)	
**Smoking**, ***n*** **(%)**				< 0.001
No	9374 (92.2)	3980 (94.6)	5394 (90.5)	
Yes	794 (7.8)	227 (5.4)	567 (9.5)	
**Drinking**, ***n*** **(%)**				0.195
No	7842 (77.1)	3273 (77.7)	4569 (76.6)	
Yes	2334 (22.9)	939 (23.4)	1395 (23.4)	
**Physical activities**, ***n*** **(%)**				0.673
No	1003 (9.9)	422 (10.0)	581 (9.8)	
Yes	9132 (90.1)	3779 (90.0)	5353 (90.2)	
**Age of menarche**, ***n*** **(%)**				0.001
≤ 15 years old	3587 (42.1)	1523 (44.2)	2064 (40.7)	
>15 years old	4929 (57.9)	1919 (55.8)	3010 (59.3)	
**Childhood health status**, ***n*** **(%)**				0.672
About average	4550 (51.4)	1891 (51.7)	2659 (51.1)	
Healthier	3169 (35.8)	1309 (35.8)	1860 (35.8)	
Less healthy	1137 (12.8)	456 (12.5)	681 (13.1)	
**Childhood socioeconomic conditions**, ***n*** **(%)**				0.715
About average	4522 (51.0)	1860 (50.8)	2662 (51.1)	
Better	869 (9.8)	370 (10.1)	499 (9.6)	
Worse	3473 (39.2)	1429 (39.1)	2044 (39.3)	

### 3.2. Prevalence of multimorbidity and the distribution of chronic diseases

**Figure 1:**
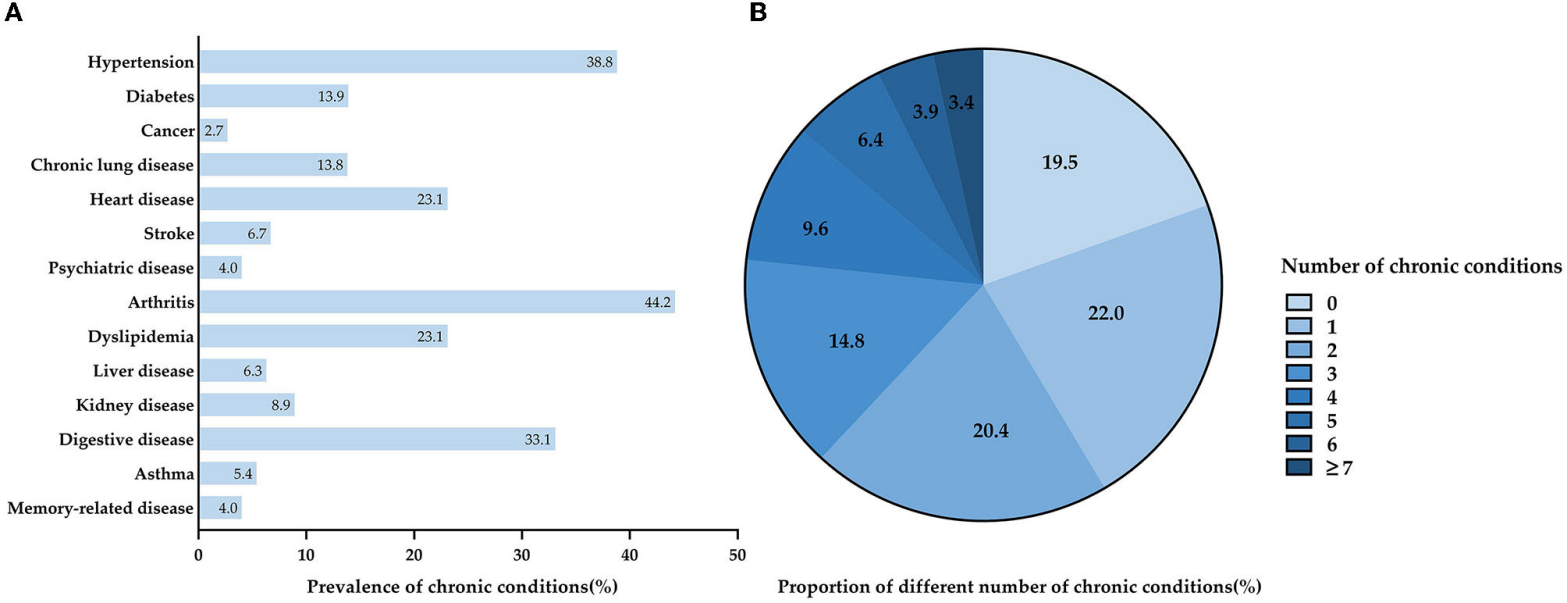
Prevalence of multimorbidity and the distribution of chronic diseases of participants. **(A)** Prevalence of chronic conditions among participants in this study. **(B)** The proportion of different numbers of chronic conditions among participants in this study.

The prevalence of multimorbidity and the distribution of chronic diseases among participants are shown in Figure 1. Of the 14 chronic conditions, the most prevalent chronic conditions were arthritis (44.2%), followed by hypertension (38.8%), and digestive disease (33.1%). Participants had an average of 2.75 chronic conditions; 19.5% of the participants had none of the chronic conditions; 22.0% had only one condition; 20.4% had two conditions, and as high as 38.1% had three or more conditions.

### 3.3. Association of female fertility history with multimorbidity and number of chronic conditions

The results of multivariable logistic regression and multivariable negative binomial regression on the association of female fertility history with multimorbidity and the number of chronic conditions are shown in Table 2. In the analysis of the association between female fertility history and multimorbidity after adjusting for covariates, the results showed that women with higher parity, including with two children (adjusted OR = 1.221, 95% CI: 1.045–1.427) and with three or more children (adjusted OR = 1.447, 95% CI: 1.215–1.723), were significantly associated with a higher risk of multimorbidity than women with only one child. In brief, women with higher parity were more likely to have multimorbidity. Age of first childbirth < 21 years (adjusted OR = 1.161, 95%CI: 1.015–1.328) was significantly associated with a higher risk of multimorbidity than the age of first childbirth ≥ 21 years. On the contrary, the age of last childbirth ≥ 35 years (adjusted OR = 0.805, 95% CI: 0.681–0.950) was significantly associated with a lower risk of multimorbidity than the age of last childbirth < 35 years.

**Table 2:** Association of female fertility history with multimorbidity and the number of chronic conditions.

**Variables**	**Multimorbidity**		**Number of chronic conditions**	
	**Unadjusted OR (95%CI)**	**Adjusted OR (95%CI)**	**Unadjusted IRR (95%CI)**	**Adjusted IRR (95%CI)**
**Parity**				
1	1.000 (Reference)	1.000 (Reference)	1.000 (Reference)	1.000 (Reference)
2	**1.257 (1.120, 1.411)**	**1.221 (1.045, 1.427)**	**1.127 (1.049, 1.210)**	**1.144 (1.041, 1.258)**
≥3	**1.940 (1.720, 2.187)**	**1.447 (1.215, 1.723)**	**1.419 (1.320, 1.528)**	**1.273 (1.147, 1.412)**
**Age of first childbirth**				
≥21 years old	1.000 (Reference)	1.000 (Reference)	1.000 (Reference)	1.000 (Reference)
< 21 years old	**1.143 (1.032, 1.265)**	**1.161 (1.015, 1.328)**	**1.082 (1.021, 1.146)**	**1.083 (1.005, 1.167)**
**Age of last childbirth**				
< 35 years old	1.000 (Reference)	1.000 (Reference)	1.000 (Reference)	1.000 (Reference)
≥35 years old	1.008 (0.894, 1.138)	**0.805 (0.681, 0.950)**	0.982 (0.917, 1.053)	**0.909 (0.828, 0.999)**

Multivariable logistic regression models and multivariable negative binomial regression models were adjusted for age, marital status, educational level, socioeconomic status, place of residence, smoking, drinking, physical activities, age of menarche, childhood health status, and childhood socioeconomic conditions.

**Figure 2:**
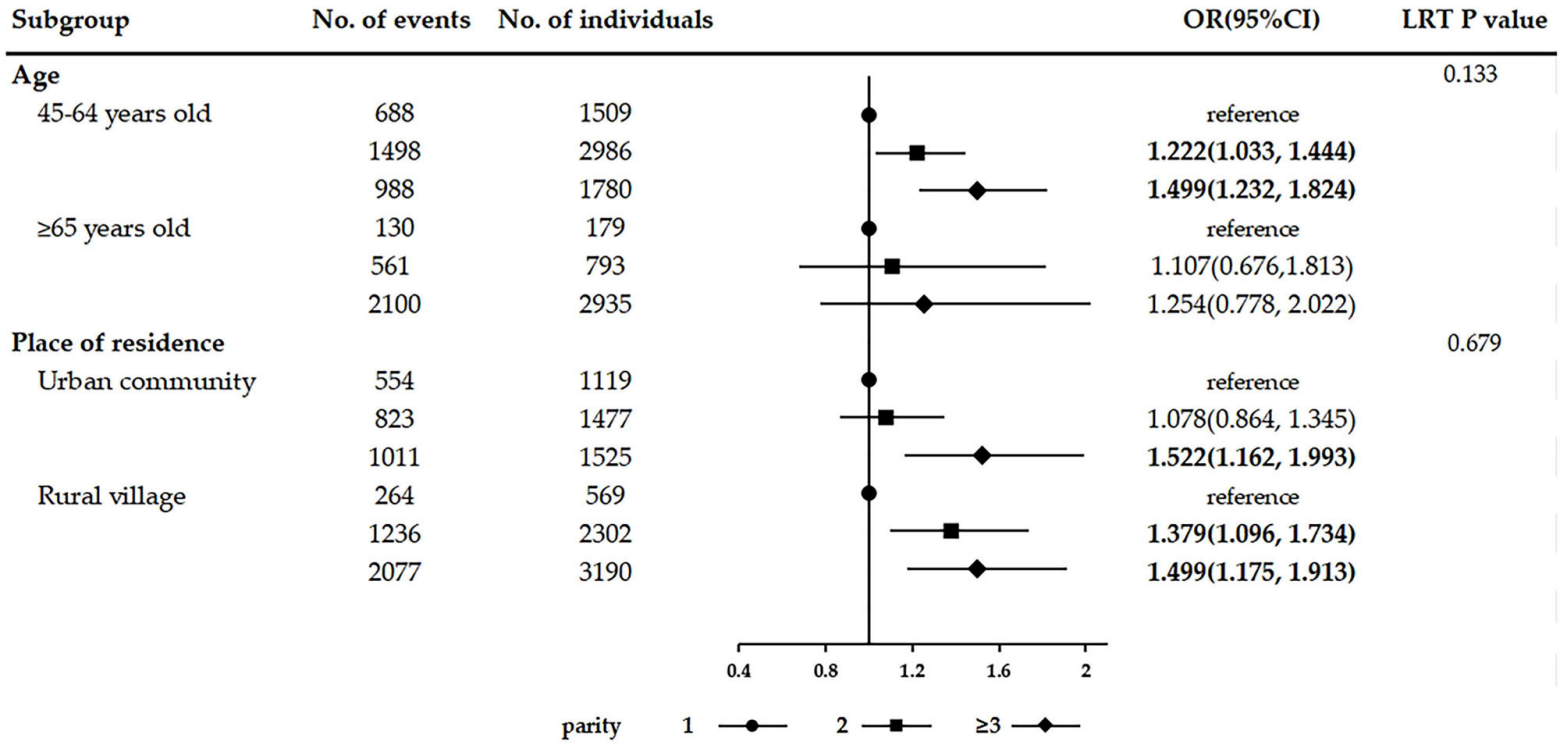
Subgroup analyses on the association between parity and multimorbidity stratified by age and place of residence.

**Figure 3:**
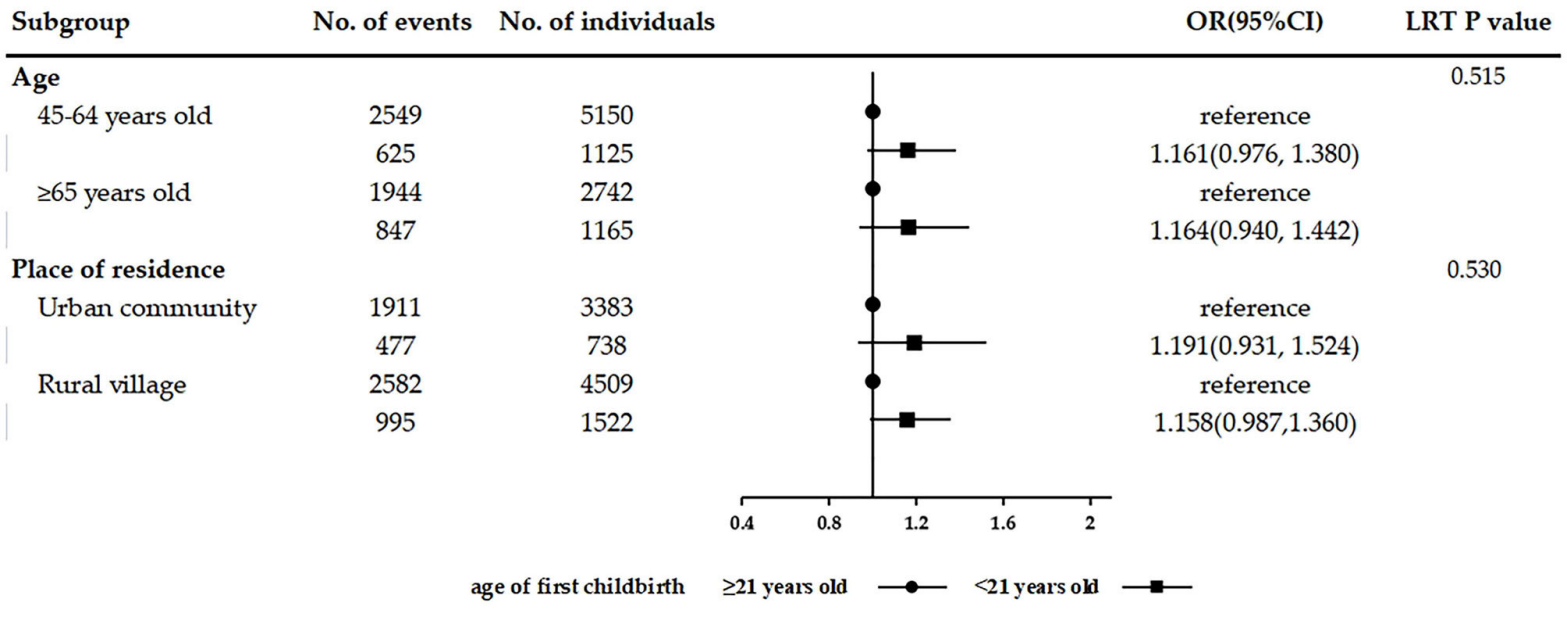
Subgroup analyses on the association between age of first childbirth and multimorbidity stratified by age and place of residence.

**Figure 4:**
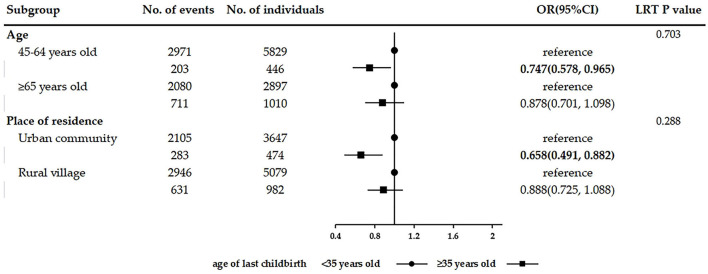
Subgroup analyses on the association between age of last childbirth and multimorbidity stratified by age and place of residence.

In the analysis of the association between female fertility history and the number of chronic conditions, the results were consistent with those observed in multivariable logistic regression after adjusting for covariates. Women with two children (adjusted IRR = 1.144, 95% CI: 1.041–1.258) and with three or more children (adjusted IRR = 1.273, 95% CI: 1.147–1.412) were significantly associated with a greater number of chronic conditions than women with only one child. Age of first childbirth < 21 years (adjusted IRR = 1.083, 95% CI: 1.005–1.167) was significantly associated with more chronic diseases than the age of first childbirth ≥21 years. Age of last childbirth ≥35 years (adjusted IRR = 0.909, 95% CI: 0.828–0.999) was observed significantly associated with fewer coexisting chronic diseases than the age of last childbirth < 35 years. The results of subgroup analyses stratified by age and place of residence are shown in Figures 2–4. The association between fertility history and multimorbidity differed between age groups and urban–rural dual structure. No evidence of interaction was found among female fertility, age, and place of residence.

**Figure 5:**
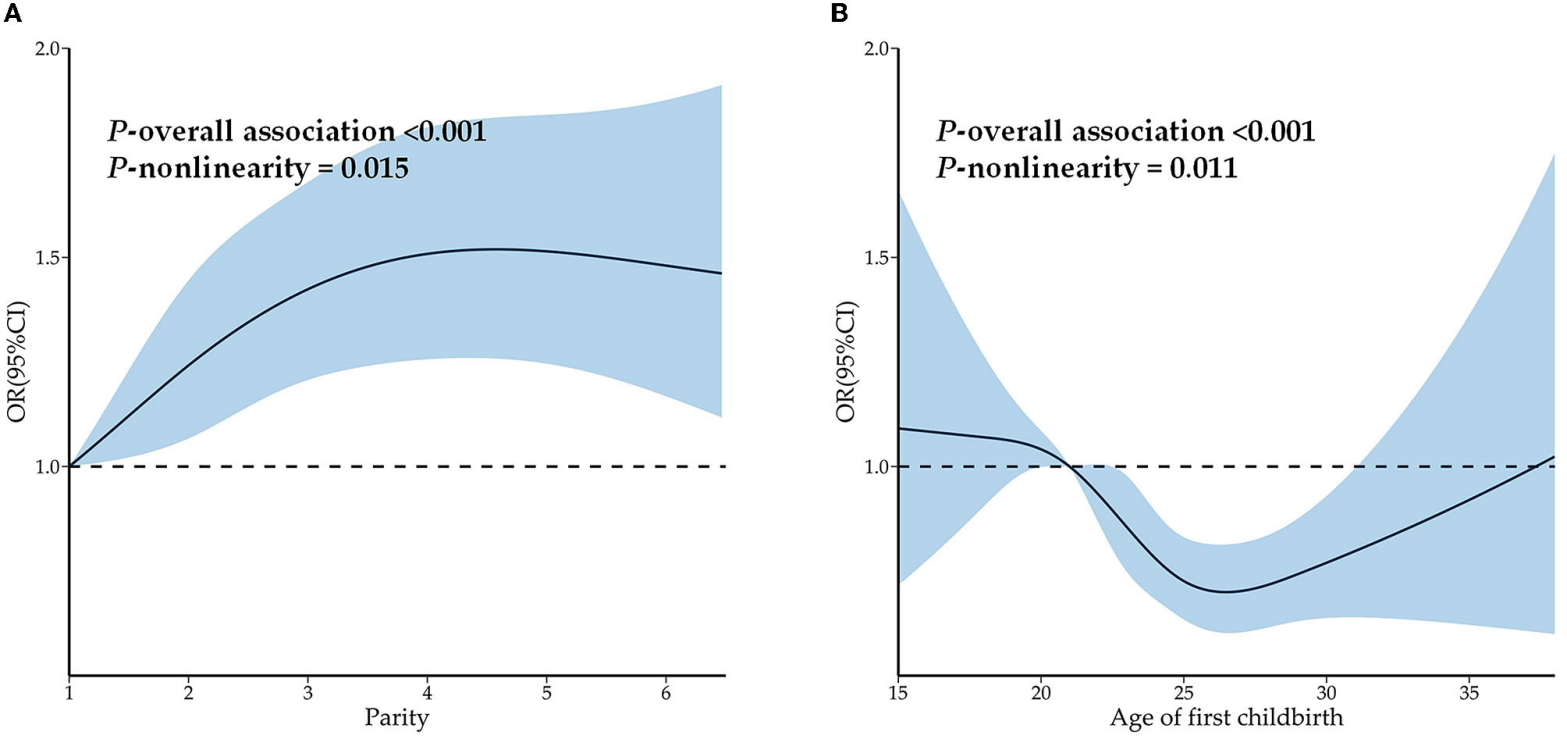
Restrictive cubic splines on the association among parity, age of first childbirth, and multimorbidity. **(A)** Restrictive cubic splines on the association between parity and multimorbidity. **(B)** Restrictive cubic splines on the association between age of first childbirth and multimorbidity.

The results of RCSs on the association among parity, age of first childbirth, and multimorbidity are shown in Figure 5. A significant nonlinear association was found between parity and odds of multimorbidity (*P*-overall association < 0.001 and *P*-non-linearity = 0.015). The odds of multimorbidity were found to increase with parity and peak when parity reached four or five. A significant nonlinear association was also observed between the age of first childbirth and odds of multimorbidity (*P*-overall association < 0.001 and *P*-non-linearity = 0.011). The odds of multimorbidity decreased with the age of first childbirth being before 26 years and increased with the age of first childbirth being after 27 years, reaching the lowest when the age of first childbirth was 26 or 27 years.

### 3.4. Association of female fertility history with multimorbidity patterns

The results of the exploratory factor analysis are shown in Table 3. Three multimorbidity patterns were identified: cardiac-metabolic pattern (dyslipidemia, hypertension, diabetes, stroke, and heart disease), visceral-arthritic pattern (digestive disease, arthritis, kidney disease, liver disease, and cancer), and respiratory-psychiatric pattern (memory-related disease, psychiatric disease, asthma, and chronic lung disease).

**Table 3:** Factor loadings of chronic conditions for multimorbidity patterns.

**Chronic conditions**	**Factor**		
	**Cardiac-Metabolic Pattern**	**Visceral-Arthritic Pattern**	**Respiratory-Psychiatric Pattern**
Dyslipidemia	0.661	0.174	−0.095
Hypertension	0.628	0.016	0.057
Diabetes	0.626	0.094	−0.114
Stroke	0.434	−0.219	0.327
Heart disease	0.416	0.289	0.169
Digestive disease	−0.013	0.638	−0.052
Arthritis	0.069	0.566	−0.060
Kidney disease	0.166	0.456	−0.027
Liver disease	0.052	0.454	−0.025
Cancer	0.079	0.211	−0.156
Memory-related disease	0.180	−0.240	0.648
Psychiatric disease	0.020	−0.114	0.588
Asthma	−0.168	0.267	0.555
Chronic lung disease	−0.149	0.415	0.470

The results of multivariable linear regression on the relationship between female fertility history and multimorbidity pattern factor scores are shown in Table 4. Having two children (adjusted β = 0.113, 95%CI: 0.038–0.188) and having three or more children (adjusted β = 0.152, 95%CI: 0.069–0.235) were positively associated with the factor scores of cardiac-metabolic pattern compared with women with only one child. Age of last childbirth ≥35 years (adjusted β = −0.136, 95%CI: from −0.212 to −0.059) was negatively associated with the factor scores of cardiac-metabolic pattern compared with the age of last childbirth < 35 years. Women with two children (adjusted β = 0.115, 95% CI: 0.038–0.191) and with three or more children (adjusted β = 0.256, 95% CI: 0.171–0.341) tend to have higher factor scores of the visceral-arthritic pattern. Age of first childbirth < 21 years (adjusted β = 0.134, 95% CI: 0.071–0.197) was positively associated with the factor scores of visceral-arthritic pattern compared with the age of first childbirth ≥21 years. For the respiratory-psychiatric pattern, women with three or more children (adjusted β = 0.132, 95% CI: 0.050–0.215) tend to have higher factor scores.

**Table 4:** Association between female fertility history and factor scores of multimorbidity patterns.

**Variables**	**Cardiac-Metabolic Pattern**	**Visceral-Arthritic Pattern**	**Respiratory-Psychiatric Pattern**
	β **(95%CI)**	β **(95%CI)**	β **(95%CI)**
**Parity**			
1	Reference	Reference	Reference
2	**0.113 (0.038, 0.188)**	**0.115 (0.038, 0.191)**	0.037 (−0.037, 0.111)
≥3	**0.152 (0.069, 0.235)**	**0.256 (0.171, 0.341)**	**0.132 (0.050, 0.215)**
**Age of first childbirth**			
≥21 years old	Reference	Reference	Reference
< 21 years old	0.020 (−0.041, 0.082)	**0.134 (0.071, 0.197)**	0.059 (−0.003, 0.120)
**Age of last childbirth**			
< 35 years old	Reference	Reference	Reference
≥35 years old	–**0.136 (**–**0.212**, –**0.059)**	−0.065 (−0.143, 0.013)	0.052 (−0.024, 0.128)

Multivariable linear regression models were adjusted for age, marital status, educational level, socioeconomic status, place of residence, smoking, drinking, physical activities, age of menarche, childhood health status, and childhood socioeconomic conditions.

**Figure 6:**
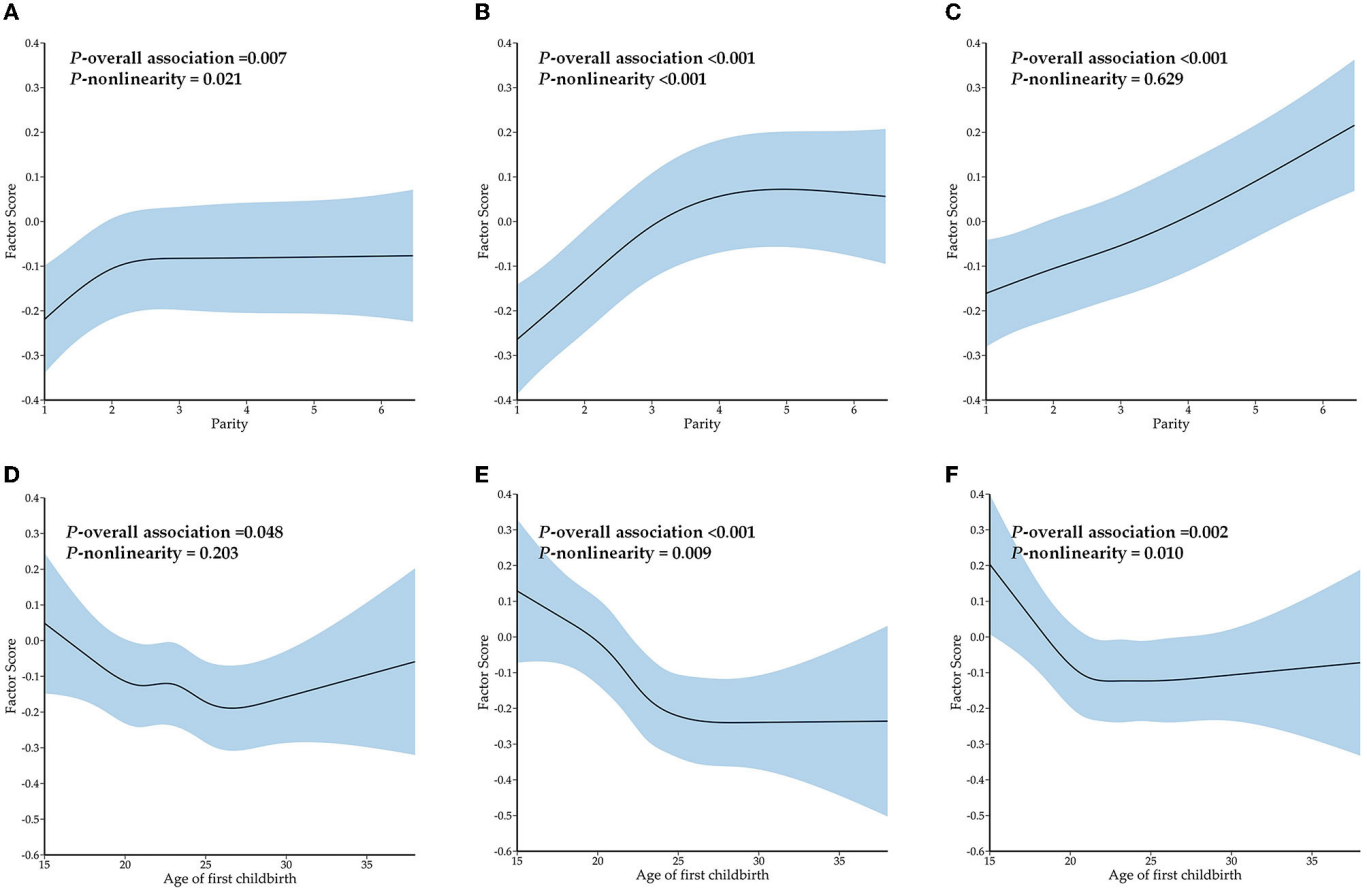
The restrictive cubic splines on the association among parity, age of first childbirth, and factor scores of multimorbidity patterns. **(A)** Restrictive cubic splines on the association between parity and factor scores of the cardiac-metabolic pattern. **(B)** Restrictive cubic splines on the association between parity and factor scores of the visceral-arthritic pattern. **(C)** Restrictive cubic splines on the association between parity and factor scores of the respiratory-psychiatric pattern. **(D)** Restrictive cubic splines on the association between age of first childbirth and factor scores of the cardiac-metabolic pattern. **(E)** Restrictive cubic splines on the association between age of first childbirth and factor scores of the visceral-arthritic pattern. **(F)** Restrictive cubic splines on the association between age of first childbirth and factor scores of the respiratory-psychiatric pattern.

The results of RCSs on the association among parity, age of first childbirth, and factor scores are shown in Figure 6. Parity had a significant non-linear association with the factor scores of the cardiac-metabolic pattern (*P*-overall association = 0.007 and *P*-non-linearity = 0.021) and visceral-arthritic pattern (*P*-overall association < 0.001 and *P*-non-linearity < 0.001) and a significant linear association with factor scores of the respiratory-psychiatric pattern (*P*-overall association < 0.001 and *P*-non-linearity = 0.629). Age of first childbirth had a significant linear association with the factor scores of the cardiac-metabolic pattern (*P*-overall association = 0.048 and *P*-non-linearity = 0.203) and a significant non-linear association with the factor scores of the visceral-arthritic pattern (*P*-overall association < 0.001 and *P*-non-linearity = 0.009) and respiratory-psychiatric pattern (*P*-overall association = 0.002 and *P*-non-linearity = 0.010). In general, parity was positively correlated with the factor scores of the three patterns, whereas the age of first childbirth was negatively correlated with the factor scores of the three patterns.

### 3.5. Sensitivity analysis

The results of the sensitivity analysis are shown in Supplementary Tables 1, 2. MICE was used to impute missing data, and hukou was used as a variable instead of residence. The results of re-analyses were consistent with the main findings of the study, and they showed the same trend.

## 4. Discussion

To the best of the authors' knowledge, this study was the first to investigate the relationship between fertility history and multimorbidity from the perspective of life courses. It was also the first study focused on the sex-specific risk factors of multimorbidity in middle-aged and elderly women in China. The results showed that high parity and early childbearing were significantly associated with multimorbidity and an increased number of chronic conditions. Late childbearing was a significant protective factor for multimorbidity and the number of chronic conditions. The association between fertility history and multimorbidity was influenced by age and urban–rural dual structure. Significant non-linear associations between parity and odds of multimorbidity and between the age of first childbirth and odds of multimorbidity were observed. Moreover, women with higher parity tended to have higher factor scores of cardiac-metabolic, visceral-arthritic, and respiratory-psychiatric patterns. Early childbearing was positively associated with the factor scores of the visceral-arthritic pattern. Late childbearing was negatively associated with the factor scores of the cardiac-metabolic pattern.

With the acceleration of the aging process, middle-aged and elderly women in China are experiencing an unprecedented burden of multimorbidity, with a prevalence rate of 58.6% in this study. Similar to other studies on the association between life course and multimorbidity (26–28), this study proved that fertility is an important event in the life course of middle-aged and elderly women in China, and its effect on multimorbidity is a promising topic worth exploring. The mechanisms underlying the association between fertility and multimorbidity may be complex including direct physiological and psychological damage and indirect socioeconomic effects (29).

Previous studies have provided evidence for the direct effects of female fertility on multimorbidity in middle and later lives, including the adverse physiological and psychological consequences. Women's abnormal life course fertility, such as high parity and early childbearing, was found to lead to undernutrition (30), frequent immune suppression (31), and metabolic disturbance (32, 33). In addition, fertility events could have a negative effect on women's mental health (34, 35). Accumulated physiological and psychological stress could lead to an increased risk of multimorbidity and an increased number of chronic diseases (36, 37). No consensus was observed on the relationship between late childbearing and health in middle and later lives (14, 21, 38). However, late childbearing in this study was found to reduce the risk of multimorbidity. This could be explained by increased exposure to endogenous estrogen as a result of a prolonged reproductive period stimulating the women's biological system positively (21). Increased endogenous estrogen was found to reduce the risk of multiple diseases such as cardiovascular diseases (39) and dementia (40). Another mechanism that underlies the relationship between fertility and multimorbidity may be socioeconomic inequalities in Chinese society during female childbearing progress (41, 42). Giving first childbirth at an early age means that young women have to play the role of a mother before they are physically and mentally mature. The lack of economic independence and the inability of Chinese society to timely provide these young mothers with financial aid and psychological counseling results in a huge cost for these women to familiarize themselves with the new roles. Families with many children force women to allocate social and health resources to their husbands or children, easily leading to resource imbalance between families and individuals (43).

The multimorbidity patterns of middle-aged and elderly women in China were identified as the cardiac-metabolic pattern, visceral–arthritic pattern, and respiratory-psychiatric pattern. Women with high parity tended to have higher factor scores of the cardiac-metabolic pattern whereas those with late childbearing tended to have lower factor scores of the cardiac-metabolic pattern. Previous studies have found similar results, that is, high parity was associated with diabetes (44), heart disease (45), and stroke (46). Giving birth at a later age was associated with lower vascular risk (47). High parity and early childbearing were positively associated with the factor scores of visceral-arthritic pattern, consistent with previous findings. High parity and early childbearing increased the risk of breast cancer (48), liver disease (49), and arthritis (50). High parity was also associated with higher factor scores of respiratory-psychiatric pattern diseases, such as asthma (51) and depression (52).

In the context of global rapid population growth, the Chinese government introduced the “late, long, few” policy in the early 1970s and an even stricter one-child policy in 1979 (53). Although the one-child policy ended in 2016, the majority of female participants in CHARLS were subjected to it during their childbearing years, which had a significant effect on Chinese women's reproductive behaviors and subsequent health outcomes in their middle and later lives. Subgroup analyses showed that the association between fertility history and multimorbidity differed between age and place of residence groups. High parity and late childbearing had a significant effect on multimorbidity in women aged 45–64 years, but not in those aged 65 years or above. This could be largely explained by aging, the strongest driver of multimorbidity. It concealed the association between fertility history and multimorbidity in elderly women. However, different childbearing policy contexts also influenced this association (54). Women aged 45–64 years were affected by one-child quotas during their child-bearing years while those aged 65 years or above had children before introducing the strict one-child policy. Under the one-child policy, women with higher parity were subjected to more pressure which adversely affects their health, while those with late childbearing could enjoy more preferential treatment to improve their health.

The dual structure of urban and rural areas in China leads to differences in economic development and fertility concepts, as well as obvious differences in the effect of these fertility restriction policies. Rural women having two or more children faced a higher risk of multimorbidity than those having only one child. However, urban women showed an increase in their risk of multimorbidity when having three or more children. Women in rural areas are not able to afford the penalties for having above-quota births, and a one-time fine could be a considerable portion of their income (55). Under the fertility restriction policy, rural women could not obtain economic support from the government, so the cost of having two children is difficult to bear. In addition, the relatively low level of medical treatment in rural areas and the backward concept of fertility lead to individuals' lower socioeconomic status, resulting in the greater effect of high parity on the multimorbidity of rural women in their middle and later years (56). Late childbearing showed a significant protective effect only among urban women. This finding may be largely explained by the fact that when urban women give birth after the age of 35 years, it is often the result of a shift in fertility decisions with higher education levels (57). Urban women in this age group enjoy better medical conditions and less labor pressure. However, rural women's late childbearing is often due to the traditional idea of “more kids, more blessings” and “preference for sons over daughters.” Rural women also suffer from malnutrition and poor reproductive healthcare, and they often perform heavy farm work to supplement family income, thus weakening the protective effect of late childbearing on the middle and later years of these women.

The results of this study are of great importance for formulating effective public health policies and programs from multiple perspectives, reducing the prevalence of multimorbidity, and improving the health of middle-aged and elderly women in China. The entire society should pay attention to women's reproductive health; strengthen the popularization of women's reproductive knowledge; change the wrong conception of fertility, such as “more kids, more blessings” and encourage women to give birth at the most appropriate age and limit excessive parity. This study showed that women who give their first childbirth at the age of 26–27 years could reduce the risk of multimorbidity in their middle and later lives. At the same time, economic support, medical assistance, and psychological counseling should be provided to women of childbearing age, especially those with a history of high parity and early childbearing. The government should trace the cumulative effect of fertility history on multimorbidity among middle-aged and elderly women, reduce their greater financial burden of coping with multimorbidity, and provide better medical services to improve the poorer clinical outcomes associated with multimorbidity. Due to the imbalance between urban and rural development, rural women should consider the subsequent greater pressure of multimorbidity. The government should redistribute health resources and increase investment in rural medical services.

This study has some limitations, so the interpretation of the results is reserved to a certain extent. First, cross-sectional data were used and not longitudinal data, leading to limitations in demonstrating causal associations between fertility history and multimorbidity. Second, data on the life course was collected mainly by questioning the participants, inevitably leading to recall bias. Finally, other life-course events, such as adverse childhood experience, were not included in this study, which could affect the strength of interpretation of the association between fertility history and multimorbidity.

## 5. Conclusion

In this study, the association between life course fertility history and multimorbidity among middle-aged and elderly women in China was evaluated. High parity and early childbearing were significantly associated with an increased risk of multimorbidity and an increased number of chronic conditions. Late childbearing was significantly associated with a lower risk of multimorbidity. Age and place of residence had a significant influence on the association between female fertility and multimorbidity. Fertility history had a significant effect on the factor scores of multimorbidity patterns. This study provided data support for exploring the sex-specific risk factors for multimorbidity and assessing the effect of life course on the prevalence of multimorbidity among middle-aged and elderly women in China. Moreover, some suggestions for reducing their burden of multimorbidity were provided.

## Data availability statement

The raw data supporting the conclusions of this article will be made available by the authors, without undue reservation.

## Ethics statement

Written informed consent was obtained from the individual(s), and minor(s)' legal guardian/next of kin, for the publication of any potentially identifiable images or data included in this article.

## Author contributions

MC, JG, and YL contributed to the study design, formal analysis, interpretation, and drafted the manuscript. JX, YH, LY, XX, LZ, JZ, and ZZ performed a formal analysis. HL, SL, and SW contributed to the design of this study, the interpretation of data, and the critical revision of the manuscript. All authors read and approved the final manuscript.
