# Supplementary material for: Life-course fertility and multimorbidity among middle-aged and elderly women in China: Evidence from China health and retirement longitudinal study
Source: Front Public Health. 2023 Feb 20;11:1090549. doi: 10.3389/fpubh.2023.1090549 (PMC9986627; doi:10.3389/fpubh.2023.1090549)
Supplement: Supplementary file 1 [file Data_Sheet_1.docx]

Supplementary Material

**Supplementary Figure 1.** The selection process of participants in this study.


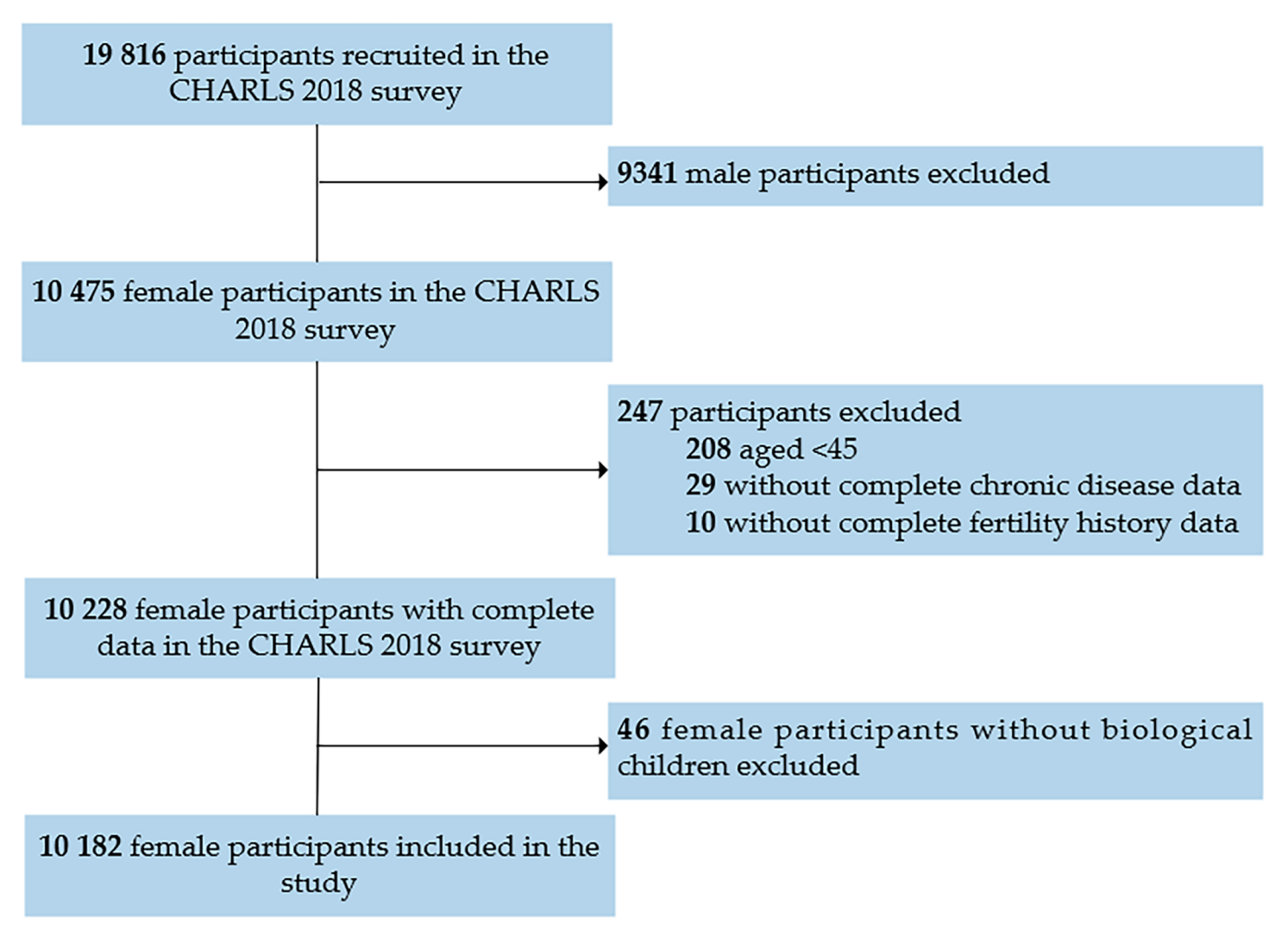


**Supplementary Table 1.** Association of female fertility history with multimorbidity and number of chronic conditions, with imputed data.

| **Variables** | **Multimorbidity** | | **Number of chronic conditions** | |
| --- | --- | --- | --- | --- |
|  | **Unadjusted OR (95%CI)** | **Adjusted OR (95%CI)** | **Unadjusted IRR (95%CI)** | **Adjusted IRR (95%CI)** |
| Parity |  |  |  |  |
| 1 | 1.000(Reference) | 1.000(Reference) | 1.000(Reference) | 1.000(Reference) |
| 2 | **1.257(1.120, 1.411)** | **1.250(1.107, 1.411)** | **1.127(1.049, 1.210)** | **1.129(1.049, 1.216)** |
| ≥3 | **1.940(1.720, 2.187)** | **1.471(1.285, 1.684)** | **1.419(1.320, 1.528)** | **1.266(1.168, 1.372)** |
| Age of first childbirth |  |  |  |  |
| ≥21 years old | 1.000(Reference) | 1.000(Reference) | 1.000(Reference) | 1.000(Reference) |
| <21 years old | **1.143(1.032, 1.265)** | 1.107(0.997, 1.229) | **1.082(1.021, 1.146)** | **1.069(1.008, 1.134)** |
| Age of last childbirth |  |  |  |  |
| <35 years old | 1.000(Reference) | 1.000(Reference) | 1.000(Reference) | 1.000(Reference) |
| ≥35 years old | 1.008(0.894, 1.138) | **0.847(0.747, 0.961)** | 0.982(0.917, 1.053) | **0.912(0.850, 0.980)** |

Multivariable logistic regression models and multivariable negative binomial regression models were adjusted for age, marital status, educational level, socioeconomic status, place of residence, smoking, drinking, physical activities, age of menarche, childhood health status and childhood socioeconomic conditions.

**Supplementary Table 2.** Association of female fertility history with multimorbidity and number of chronic conditions adjusted with hukou.

| **Variables** | **Multimorbidity** | | **Number of chronic conditions** | |
| --- | --- | --- | --- | --- |
|  | **Unadjusted OR (95%CI)** | **Adjusted OR (95%CI)** | **Unadjusted IRR (95%CI)** | **Adjusted IRR (95%CI)** |
| Parity |  |  |  |  |
| 1 | 1.000(Reference) | 1.000(Reference) | 1.000(Reference) | 1.000(Reference) |
| 2 | **1.257(1.120, 1.411)** | **1.172(1.003, 1.370)** | **1.127(1.049, 1.210)** | **1.128(1.026, 1.241)** |
| ≥3 | **1.940(1.720, 2.187)** | **1.365(1.145, 1.628)** | **1.419(1.320, 1.528)** | **1.248(1.124, 1.386)** |
| Age of first childbirth |  |  |  |  |
| ≥21 years old | 1.000(Reference) | 1.000(Reference) | 1.000(Reference) | 1.000(Reference) |
| <21 years old | **1.143(1.032, 1.265)** | **1.156(1.011, 1.322)** | **1.082(1.021, 1.146)** | **1.081(1.003, 1.165)** |
| Age of last childbirth |  |  |  |  |
| <35 years old | 1.000(Reference) | 1.000(Reference) | 1.000(Reference) | 1.000(Reference) |
| ≥35 years old | 1.008(0.894, 1.138) | **0.807(0.683, 0.953)** | 0.982(0.917, 1.053) | **0.909(0.828, 0.999)** |

Multivariable logistic regression models and multivariable negative binomial regression models were adjusted for age, marital status, educational level, socioeconomic status, hukou, smoking, drinking, physical activities, age of menarche, childhood health status and childhood socioeconomic conditions.
